# Supplementary material for: A comparative study on the intensity of loneliness among Kenyan youth in school and home environments
Source: Glob Ment Health (Camb). 2026 Mar 10;13:e66. doi: 10.1017/gmh.2026.10172 (PMC13112292; doi:10.1017/gmh.2026.10172)
Supplement: Mutiso et al. supplementary material [file S2054425126101721sup001.docx]

| **Supplementary Table 1: Association between loneliness and marital status stratified by age group** | | | | | |
| --- | --- | --- | --- | --- | --- |
| **Loneliness scores at home aged (14-18)** | | | | | |
|  | Sum of Squares | df | Mean Square | F | Sig. |
| Between Groups | 854.284 | 6 | 142.381 | 1.02 | 0.412 |
| Within Groups | 47588.35 | 341 | 139.555 |  |  |
| Total | 48442.63 | 347 |  |  |  |
| **Loneliness scores at home aged (19-25)** | | | | | |
|  | Sum of Squares | df | Mean Square | F | Sig. |
| Between Groups | 2685.712 | 6 | 447.619 | 2.898 | **0.008** |
| Within Groups | 224614.8 | 1454 | 154.481 |  |  |
| Total | 227300.5 | 1460 |  |  |  |
| ***Note: Loneliness at school was non-significant*** | | | | | |

| **Supplementary Table 2: Interactions between gender and social demographic and their association with loneliness** | | | | | | |
| --- | --- | --- | --- | --- | --- | --- |
|  | ***Loneliness scores at home*** | | | ***Loneliness scores at school*** | | |
| **Cases** | **Mean Square** | **F** | **p** | **Mean Square** | **F** | **p** |
| Gender | 856.1 | 5.663 | **0.017** | 293.57 | 1.901 | 0.168 |
| Age group | 191.75 | 1.268 | 0.26 | 12.69 | 0.082 | 0.774 |
| Gender ✻ Age group | 79.18 | 0.524 | 0.469 | 541.5 | 3.507 | 0.061 |
| Gender | 377.3 | 2.5 | 0.114 | 147.6 | 0.952 | 0.33 |
| Marital status | 511.1 | 3.386 | **0.003** | 265.7 | 1.713 | 0.114 |
| Gender ✻ Marital status | 336.7 | 2.231 | **0.038** | 146.2 | 0.943 | 0.463 |
| Gender | 685.9 | 4.57 | **0.033** | 86.03 | 0.556 | 0.456 |
| Education | 1153.4 | 7.686 | **< .001** | 312.09 | 2.019 | 0.11 |
| Gender ✻ Education | 158.5 | 1.056 | 0.367 | 264.04 | 1.708 | 0.164 |
| Gender | 962.3 | 6.382 | **0.012** | 1941.9 | 12.609 | **< .001** |
| Employment | 296.3 | 1.965 | 0.117 | 242.3 | 1.573 | 0.194 |
| Gender ✻ Employment | 441.5 | 2.928 | **0.033** | 391.3 | 2.541 | 0.055 |
| Gender | 53.21 | 0.351 | 0.553 | 14.1 | 0.091 | 0.763 |
| Religion | 163.28 | 1.078 | 0.357 | 82.5 | 0.531 | 0.661 |
| Gender ✻ Religion | 262.28 | 1.732 | 0.158 | 302.99 | 1.951 | 0.12 |
| ***Note. Type III Sum of Squares*** | | | | | | |

| **Supplementary Table 3. Generalized Estimating Equation (GEE) Model Predicting Loneliness Score across School and Home settings.** | | | | | | |
| --- | --- | --- | --- | --- | --- | --- |
| **Predictor** | **B** | **SE** | **95% CI (Lower – Upper)** | **Wald χ²** | **df** | **p** |
| **Intercept** | 22.698 | 2.526 | 17.748 – 27.649 | 80.753 | 1 | **<0.001** |
| **Loneliness (Home vs School)** | -3.726 | 2.433 | -8.494 – 1.041 | 2.346 | 1 | 0.126 |
| **Age Group (14-18 vs 19-25)** | -1.725 | 0.799 | -3.290 – -0.159 | 4.662 | 1 | **0.031** |
| **Gender (Male vs Female)** | 2.009 | 0.604 | 0.825 – 3.194 | 11.063 | 1 | **0.001** |
| **Religion** |  |  |  |  |  |  |
| Protestant vs Others | 0.493 | 1.234 | -1.926 – 2.911 | 0.159 | 1 | 0.69 |
| Catholic vs Others | -0.376 | 1.243 | -2.811 – 2.059 | 0.092 | 1 | 0.762 |
| Muslim vs Others | -1.499 | 1.783 | -4.994 – 1.995 | 0.707 | 1 | 0.4 |
| **Employment** | |  |  |  |  |  |
| No, but volunteering | -1.752 | 1.256 | -4.215 – 0.710 | 1.946 | 1 | 0.163 |
| Full-time | -0.913 | 1.514 | -3.881 – 2.055 | 0.364 | 1 | 0.546 |
| Part-time | -0.478 | 1.896 | -4.194 – 3.239 | 0.063 | 1 | 0.801 |
| **Education** |  |  |  |  |  |  |
| Primary | 3.993 | 1.55 | 0.954 – 7.032 | 6.634 | 1 | **0.01** |
| Secondary | 1.285 | 1.251 | -1.166 – 3.736 | 1.056 | 1 | 0.304 |
| Tertiary | -1.21 | 1.277 | -3.713 – 1.292 | 0.899 | 1 | 0.343 |
| **Marital Status** | |  |  |  |  |  |
| In a relationship | -0.041 | 1.822 | -3.612 – 3.530 | 0.001 | 1 | 0.982 |
| Married/cohabiting | 0.256 | 1.943 | -3.552 – 4.065 | 0.017 | 1 | 0.895 |
| Separated | 3.407 | 2.678 | -1.840 – 8.655 | 1.619 | 1 | 0.203 |
| Divorced | 0.609 | 2.949 | -5.170 – 6.389 | 0.043 | 1 | 0.836 |
| Widowed | -3.975 | 4.969 | -13.713 – 5.763 | 0.640 | 1 | 0.424 |
| **Interactions** |  |  |  |  |  |  |
| Loneliness * Employment | | |  |  |  |  |
| Home * No, but volunteering | 3.488 | 1.311 | 0.919 – 6.057 | 7.08 | 1 | **0.008** |
| Home * Full-time | 4.906 | 1.625 | 1.722 – 8.090 | 9.12 | 1 | **0.003** |
| Home * Part-time | 3.537 | 1.999 | -0.382 – 7.456 | 3.130 | 1 | **0.077** |
| Other interactions (Loneliness * Age, Gender, Religion, Education, Marital Status) | Not significant | |  |  |  |  |
| Dependent variable: Loneliness score. The model included 2,835 observations from 1,748 participants (71.9% of the total sample). Reference categories were: Loneliness = School, Age Group = 19-25, Gender = Female, Education = University, Marital Status = Other, and Employment = Not working. Non-significant main effects included Loneliness, Religion, Employment, other Education levels, and all other interactions (p > 0.05). The working correlation structure was exchangeable, with an identity link function and a normal distribution. Model fit statistics were QIC = 427,051.80 and QICC = 427,059.83, with lower values indicating better fit. The scale parameter was 152.55. For the marital status, the overall Wald test for the factor was significant (χ² = 11.2, p = 0.035), indicating differences across categories relative to the reference group (‘never married’). The interaction between loneliness place and employment was significant overall (Wald χ² = 9.3, p = 0.023). Coefficients (B) represent mean differences in loneliness scores on a 0-60 scale. For example, B = 2.0 corresponds to an expected two-point higher loneliness score compared to the reference category. | | | | | | |
